# Occurrence of Lagovirus europaeus (Rabbit Hemorrhagic Disease Virus) in Domestic Rabbits in Southwestern Poland in 2019: Case Report

**DOI:** 10.1128/spectrum.02298-22

**Published:** 2022-11-29

**Authors:** Rafał Hrynkiewicz, Dominika Bębnowska, Ari Kauppinen, Tuija Gadd, Tomasz Piasecki, Paulina Niedźwiedzka-Rystwej

**Affiliations:** a Institute of Biology, University of Szczecin, Szczecin, Poland; b Finnish Food Authority, Laboratory and Research Division, Animal Health Diagnostic Unit, Helsinki, Finland; c Department of Epizootiology and Clinic of Birds and Exotic Animals, The Faculty of Veterinary, Wroclaw University of Environmental and Life Sciences, Wrocław, Poland; University of Prince Edward Island

**Keywords:** *Lagovirus europaeus*, RHDV, RHDVa, phylogenetic analysis, rabbit hemorrhagic disease

## Abstract

Lagovirus europaeus (rabbit hemorrhagic disease virus [RHDV]) is a small, nonenveloped, single-stranded RNA virus that causes a severe, highly infectious, and fatal disease in rabbits (Oryctolagus cuniculus) called rabbit hemorrhagic disease (RHD). Since its discovery in the 1980s, it has posed a very serious threat to the global rabbit industry and the rabbit population in the wild. According to data from 2005 to 2018, the occurrence of RHD has been reported or suspected in 50 countries, with more than one-half of the reports being recorded in European countries. The main aim of the study was to detect Lagovirus europaeus (RHDV) strains found in domestic rabbits that died suddenly in the city of Wrocław in southwest Poland. All animals (*n* = 14) tested in this study died naturally and showed macroscopic features at necropsy that indicated the possibility of death from RHD. As a result of the research, the presence of L. europaeus virus was confirmed in 8 samples of all 14 samples collected. All strains of Lagovirus europaeus isolated in the present study showed 100% nucleotide identity to L. europaeus GI.1 strain FRG and a strain isolated in New Zealand, as well as the L. europaeus GI.1a Erfurt strain. This suggests that it is likely that L. europaeus GI.2 strains have so far not displaced L. europaeus GI.1 strains from the environment in Poland.

**IMPORTANCE** Lagovirus europaeus (RHDV) causes a severe, highly infectious, and fatal disease in rabbits called RHD. The disease is a very serious threat to the global rabbit industry and the rabbit population in the wild. The aim of the study was to detect Lagovirus europaeus (RHDV) strains in domestic rabbits that died suddenly in Poland. The presence of RHDV was confirmed in 8 samples of all 14 samples collected. This is one of the very few reports on the existence of this virus in pet rabbits in Poland.

## INTRODUCTION

Lagovirus europaeus (rabbit hemorrhagic disease virus [RHDV]) is a virus that causes a severe, highly infectious, and deadly disease in rabbits (Oryctolagus cuniculus) called rabbit hemorrhagic disease (RHD) (1–3). The disease is characterized by changes in the liver and disseminated intravascular coagulation (DIC); in terms of its etiology, RHD is similar to acute viral hepatitis (4–7).

L. europaeus is a small, nonenveloped, single-stranded RNA (ssRNA) positive-strand virus (1, 3, 8) belonging to the *Caliciviridae* family and the *Lagovirus* genus (9). Since the discovery of this pathogen in the 1980s (10), it has posed a very serious threat to the global rabbit industry and the rabbit population living in the wild (2, 3). This situation is due to the very high genetic variability among the circulating virus strains (2, 3, 11, 12).

The *Lagovirus* genus has been divided into two main genogroups, namely, the GI and GII genogroups. The GI group is represented by RHD virus (RHDV), while the GII group is represented by the European brown hare syndrome virus (EBHSV) (1). Within the GI genogroup, four genotypes have been distinguished, i.e., the GI.1, GI.2, GI.3, and GI.4 genotypes (1). The GI.1 genotype includes the classic RHDV strains (L. europaeus GI.1), while the GI.2 genotype has been classified as RHDV2 (L. europaeus GI.2) (1). The GI.3 genotype is rabbit calicivirus E1 (RCV-E1) (L. europaeus GI.3), while the GI.4 genotype is represented by RCV-A1 (L. europaeus GI.4) and RCV-E2 (L. europaeus GI.4d) (1, 13–15). Strains classified into the GI.3 and GI.4 genotypes are termed nonpathogenic RCV. The GI.1 and GI.2 genotype strains are known infectious agents responsible for the development of RHD (1, 2). In the GI.1 genotype, four antigenic variants were distinguished on the basis of phylogenesis and genetic distance and, according to the new nomenclature, were designated GI.1a, GI.1b, GI.1c, and GI.1d (1).

The first mention of L. europaeus GI.1 (RHDV) was made in 1984 in Wuxi, Jiangsu Province, China (10). The disease was observed in a population of European rabbits (O. cuniculus) of the Angora breed that had been imported from the former German Democratic Republic to China for breeding purposes (2, 3, 10). In China, in 1 year alone, the disease contributed to the deaths of as many as 140 million domestic rabbits and spread over approximately 50,000 km^2^, causing enormous damage to the country's economy (2, 16). In a very short time, the virus has spread throughout the world and is threatening the national economies of countries that, for the most part, rely on the rabbit industry for their economies (12). The disease is now established in Europe, Asia, Africa, and Australia (4, 5, 17). All outbreaks of RHD until 2010 were caused by L. europaeus strains of genotype GI.1 (RHDV) and its antigenic variants (GI.1a to GI.1d) (12). A new strain of L. europaeus GI.2 that was originally identified as RHDV2 (18) was confirmed in France in 2010 and, like the classic strains of RHDV, spread worldwide very rapidly (18, 19). To date, no effective cure for RHD that can save infected rabbits has been invented, and the only way to fight the disease is preventive vaccination (11, 20, 21). Currently, several vaccines that are effective against both strains of L. europaeus are available in the world (12).

Because of its severe course, high infectivity, and high mortality rate and because of the high risk and large financial losses for the livestock industry (2, 3, 11), RHD is included in the list of diseases of the World Organisation for Animal Health (WOAH) (founded as the Office International des Epizooties [OIE]) and is subject to compulsory notification of every case (22). An animal suspected or confirmed to have Lagovirus europaeus is immediately euthanized (22, 23). According to data from 2005 to 2018, the occurrence of RHD was reported or suspected in 50 countries, with more than one-half of the reports being from European countries (23).

The aim of the study was to detect strains of Lagovirus europaeus (RHDV) present in domestic rabbits that died suddenly in 2019 in the city of Wrocław in southwestern Poland.

## RESULTS

### RT-qPCR.

The results of the detection of L. europaeus GI.1 and L. europaeus GI.2 viruses by reverse transcription-quantitative PCR (RT-qPCR) are presented in Table 1.

**TABLE 1:** Results of RT-qPCR for detection of RHDV and RHDV2 in liver tissue samples

Sample no.	RHDV probe results			RHDV2 probe results		
	Result	*T_m_* (°C)	*C_T_* ^*a*^	Result	*T_m_* (°C)	*C_T_*
WR/1	Positive	86.50	36.12	Negative		
WR/2	Positive	86.36	34.18	Negative		
WR/3	Negative			Negative		
WR/5	Negative			Negative		
WR/6	Negative			Negative		
WR/7	Negative			Negative		
WR/8	Positive	86.32	33.19	Negative		
WR/9	Negative			Negative		
WR/12	Positive	86.08	34.08	Negative		
WR/13	Positive	86.07	30.87	Negative		
WR/14	Positive	85.79	32.81	Negative		
WR/15	Negative			Negative		
WR/17	Positive	85.99	33.05	Negative		
WR/20	Positive	85.94	31.71	Negative		
Negative control for RHDV	Negative			Negative		
Negative control for RHDV2	Negative			Negative		

a *C_T_*, threshold cycle.

Fourteen liver tissue samples, taken directly from animals that had died naturally, were tested. All liver tissue samples were tested for L. europaeus GI.1 and L. europaeus GI.2. In the case of L. europaeus GI.1 strains, 8 positive results were obtained, from individuals WR/1, WR/2, WR/8, WR/12, WR/13, WR/14, WR/17, and WR/20. The remaining 6 animals were negative for the presence of L. europaeus GI.1. The tests for L. europaeus GI.2 did not confirm the presence of the strains sought.

Analysis of the melting temperature (*T_m_*) values obtained for all strains showed that all results were similar, ranging from 85.79°C to 86.50°C (Table 1). The *T_m_* values for all tested samples exclude the possibility of a false-negative result due to the formation of nonspecific reaction products. To confirm the results obtained by RT-qPCR, all tested samples were sequenced.

Table 2 shows the results of the analysis for absolute quantification. The virus copy numbers were tested in 8 positive samples and are expressed as RNA copies per milligram of liver.

**TABLE 2:** Analysis results for absolute quantification

Sample no.	Animal identification no.	No. of RNA copies/mg liver
1	WR/1	3.70 × 10^3^
2	WR/2	2.21 × 10^3^
3	WR/8	4.56 × 10^3^
4	WR/12	6.67 × 10^4^
5	WR/13	3.11 × 10^3^
6	WR/14	1.10 × 10^4^
7	WR/17	2.38 × 10^4^
8	WR/18	2.92 × 10^3^

### Sequencing and phylogenetic analysis.

**FIG 1:**
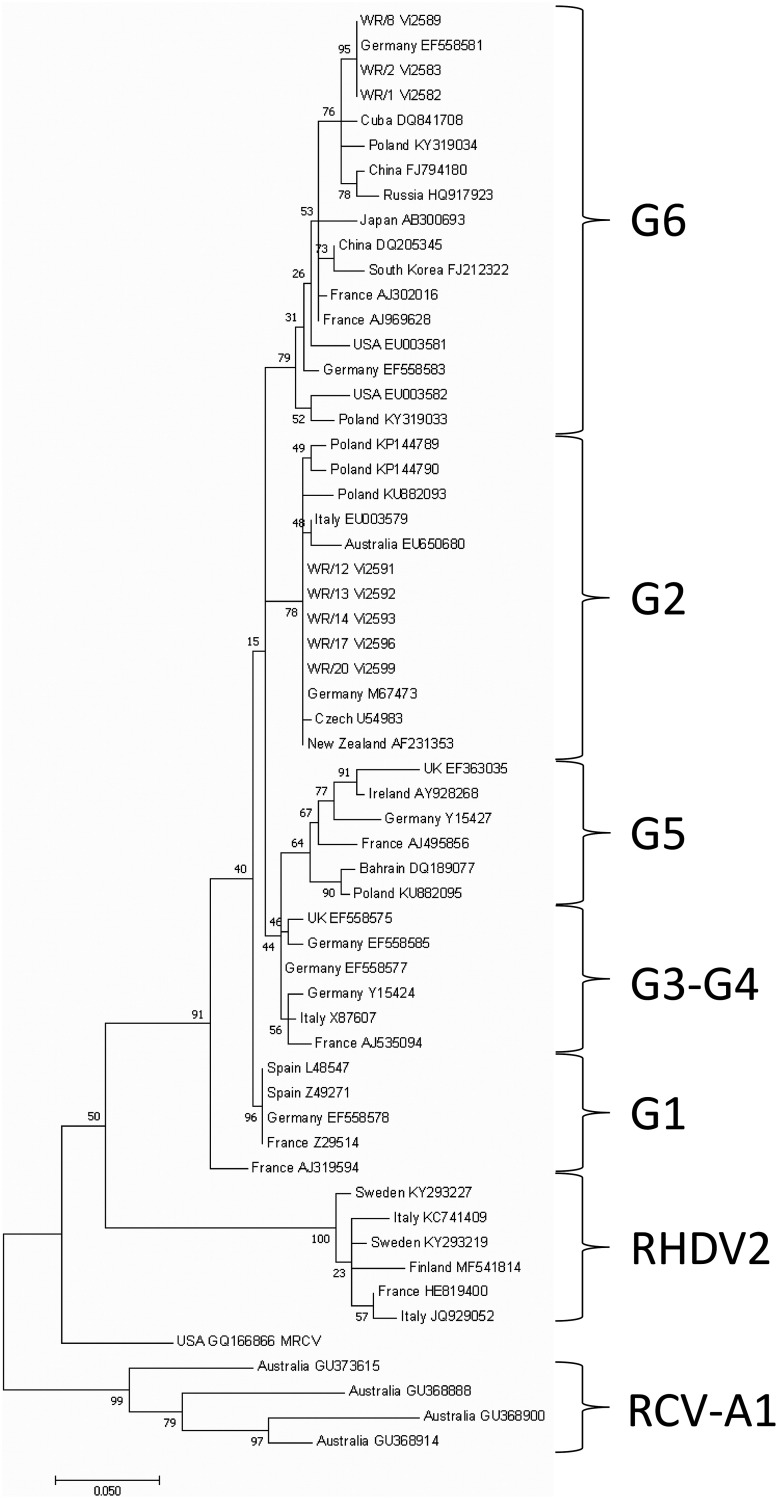
Phylogenetic tree based on partial sequences of the VP60 capsid protein gene of representative RHDV isolates. The genetic groups of RHDV are indicated with curly brackets. The country of origin and the GenBank accession number are given for previously published RHDV sequences. The numbers at the nodes of the tree indicate bootstrap values of 1,000 replicates; values under 70 are not shown.

Sequencing of the partial VP60 gene revealed that strains isolated from samples WR/1, WR/2, and WR/8 were identical and grouped into the GI.1a genogroup, sharing 100% nucleotide identity with the Erfurt strain (GenBank accession number EF558581). Similarly, strains isolated from samples WR/12, WR/13, WR/14, WR/17, and WR/20 were identical and grouped into the GI.1c genogroup, sharing 100% nucleotide identity with strain FRG (GenBank accession number M67473) and a strain isolated in New Zealand (GenBank accession number AF231353). The two groups of samples (GI.1a and GI.1c) isolated in this study shared nucleotide identity of 93.9% (263/280 nucleotides) (Fig. 1).

## DISCUSSION

Lagovirus europaeus causes RHD, which is a highly fatal disease that occurs in wild and domestic rabbits and other lagomorphs (3). The disease can occur in three clinical forms, namely, hyperacute, acute, and subacute. In the hyperacute form, sudden death is observed among animals that show little or no signs of the disease. In the acute form, the symptoms are much more severe; this is also observed in the subacute form, for which, however, the survival rate is much higher and past disease determines the occurrence of natural protection against reinfection (2, 3). Disease symptoms include neurological signs (e.g., agitation, seizures, and ataxia) and gastrointestinal disturbances, but respiratory distress may also be present. The histopathological picture shows multiple lesions in multiple organs but most notably acute hepatitis and splenic enlargement. Congestion, hemorrhage, and embolism due to massive DIC can be observed in many organs (2, 3).

All of the animals we analyzed in our study died naturally and at necropsy showed macroscopic features that indicated the possibility of death due to RHD. However, a major limitation is that thorough histopathological examinations were not performed during the procedures, and thus the description of the pathological features in the rabbits analyzed is not very accurate. In some cases, data are incomplete or there is a complete lack of information. In a study by Harcourt-Brown et al. (24), it was noted that, if a necropsy is performed 12 to 24 h after the animal's death, then the results can be misleading due to autolysis of rabbit tissues, despite the carcass being kept in a refrigerator. In the case of our work, we cannot unequivocally determine the time between the death of the animal and the performance of the necropsy.

The liver is one of the target sites for virus replication; therefore, we determined the presence of L. europaeus GI.1 and GI.2 in liver sections collected from all dead animals by real-time PCR. As a result, we confirmed the presence of the virus in 8 samples tested, with the GI.1 genotype but not the GI.2 genotype being detected in all samples. In some cases, RHD features in the liver were poorly expressed macroscopically, and so we additionally quantified the number of viral RNA copies per milligram of liver. In their study, Harcourt-Brown et al. (25) reported that PCR results are complementary to histopathological examination results in the diagnosis of RHD, and attention should be paid to the possibility of false-negative results.

RHD was first detected in 1984 in China (10). The first reports of rabbit infection with L. europaeus virus (RHDV) in Poland appeared in 1988 (26). Currently, the presence of three pathogenic forms of L. europaeus virus (RHDV), i.e., GI.1, GI.1a, and GI.2, in Poland has been confirmed (27). In 2003, Fitzner and Kęsy (28) presented their study in which a phylogenetic analysis of Polish L. europaeus (RHDV) strains, i.e., SGM 1988, KGM 1988, LUB 1988, PD 1989, MAL 1994, BLA 1994, GSK 1988, and ZD0 2000, which were collected between 1988 and 2000, was performed for the first time (27). Both nucleotide and amino acid sequences of Polish L. europaeus (RHDV) strains showed high genetic identity of the isolates and, on this basis, two genetic groups showing temporal similarity were determined (26, 27). Research presented in 2012 by Fitzner et al. (29), in which 15 Polish L. europaeus (RHDV) strains isolated over 18 years were analyzed, confirmed the presence of three genetic groups. The oldest Polish strain of L. europaeus (RHDV) showed very high similarity at the amino acid level (98 to 99%) to the German reference strain FRG89 and most virus strains isolated in Europe during the same period, as well as to the Chinese isolate from 1984 (27, 28). Similarly, the analysis of strains isolated in Poland in 2012 to 2016 showed the presence of classic RHDV strains from three genogroups (BLA 1994, OPO 2004, GSK 1988, and ZD0 2000) and strains belonging to the antigenic variant (GRZ 2004, KRY 2004, L145 2004, W147 2005, SKO 2013, GLE 2013, RED1 2013, STR 2012, STR2 2013, STR 2014, and BIE 2015) (27). The analyses also confirmed the disappearance of the classic RHDV and the domination of the antigenic variant in Poland (27). In 2018, Fitzner and Niedbalski (30) reported the first Polish isolates of L. europaeus GI.2 (RED 2016 and VMS 2017). The genetic tests conducted with isolates from domestic rabbits in 2018 showed the presence of L. europaeus GI.2 strains (PIN, LIB, and WAK). In wild rabbits, the presence of a L. europaeus GI.1a strain (F77-3) was demonstrated in 2015 (31). In our study, we confirmed the presence of Lagovirus europaeus in 8 domestic rabbits that suddenly died in 2019 in Wrocław, in southwest Poland. Genetic studies of the isolates showed that the three isolates (WR/1, WR/2, and WR/8) were identical and clustered in the GI.1a genogroup and showed 100% nucleotide identity with the German strain Erfurt. The Lagovirus europaeus strains isolated from samples WR/12, WR/13, WR/14, WR/17, and WR/20 were identical, as in the case of the previous three strains, but were grouped in genogroup GI.1c and showed 100% nucleotide identity with the German strain FRG and a strain isolated in New Zealand. The two groups of samples (GI.1a and GI.1c) isolated in this study showed nucleotide identity of 93.9% (263/280 nucleotides).

### Conclusion.

The Lagovirus europaeus strains we isolated from pet rabbits showed 100% nucleotide identity with L. europaeus GI.1strain FRG and a strain isolated in New Zealand or L. europaeus GI.1a strain Erfurt. This finding suggests that L. europaeus GI.2 probably did not displace L. europaeus GI.1 strains from the environment in Poland.

## MATERIALS AND METHODS

### Samples.

Tissue samples were collected in 2019 from 14 pet rabbits with suspected Lagovirus europaeus (RHDV) infection. All tissue samples were collected within the city of Wrocław in southwestern Poland. Each animal, immediately after demise, was reported and directly transported to the Veterinary Clinic Zwierzyniec (Diagnostic and Treatment Centre for Exotic Animals), where they underwent necropsy. All tissues were described in detail, and animals were given a number with the prefix WR and an identification number. For all animals, postmortem imaging showed characteristic changes indicative of Lagovirus europaeus (RHDV) infection. Table 3 shows the postmortem descriptions of the animals, along with their sex, age, and current vaccination status.

**TABLE 3:** Postdissection image changes, sex, age, and current vaccination status of the tested animals

Sample no.	Animal identification no.	Sex	Age^*a*^	Changes in dissection image	Symptoms before fall of animal	Vaccination status
1	WR/1	Female	6 yr	Trachea: severe hyperemia; lungs: severe swelling; liver: swollen, pale	Gastrointestinal problems	Unvaccinated
2	WR/2	Female	7 yr	Trachea: hyperemia; lungs: edema, severe congestion; liver: pale, brittle	Sudden breathlessness, agonizing state	Unvaccinated
3	WR/3	Female	13 wk	Trachea: slight hyperemia; lungs: swelling, congestion; liver: fatty degeneration	Runny nose, shortness of breath	Unvaccinated
4	WR/5	Female	6.5 yr	Trachea: foamy bloody liquid; lungs: petechiae, swollen; liver: unchanged	Bloody urine, nystagmus, severe shortness of breath	No current vaccinations
5	WR/6	Male	ND	Trachea: unchanged; lungs: unchanged; liver: fatty degeneration	Dental patient	ND
6	WR/7	Male	ND	Trachea: unchanged; lungs: very severe swelling, ecchymosis; liver: unchanged	ND	ND
7	WR/8	Female	ND	Trachea: severe hyperemia; lungs: severe congestion, swelling; liver: pale, brittle	Breathlessness, sudden death	ND
8	WR/9	Male	8.5 yr	Trachea: unchanged; lungs: unchanged; liver: unchanged	Dental patient; severe shortness of breath	ND
9	WR/12	Male	ND	Trachea: severe hyperemia; lungs: severe swelling; liver: brittle, disintegrating	ND	ND
10	WR/13	Female	ND	Trachea: severe hyperemia; lungs: severe swelling, hyperemia; liver: pale, brittle	Sudden death	ND
11	WR/14	Male	4 yr	Trachea: severe hyperemia, ecchymosis; lungs: very severe swelling, hyperemia; liver: hyperemia, brittle, disintegrating	Severe dyspnea and urinary incontinence; decision was made to euthanize	Unvaccinated
12	WR/15	Male	4 yr	Trachea: unchanged; lungs: unchanged; liver: unchanged	Dental patient; problems with digestive system	Unvaccinated
13	WR/17	Female	ND	Trachea: hyperemia; lungs: severe swelling, hyperemia; liver: brittle, disintegrating	ND	ND
14	WR/20	Male	ND	Trachea: very severe hyperemia; lungs: severe swelling, ecchymosis; liver: hyperemia, brittle	Sudden death	ND

a ND, no data.

### Isolation of viral RNA.

Total RNA was extracted from 30 mg of each liver sample using the ExtractMe total RNA kit (Blirt S.A, Gdańsk, Poland) according to the manufacturer's instructions.

### RT and cDNA synthesis.

The complementary strand of DNA (cDNA) was obtained by RT with a previously prepared viral RNA template. The reaction was performed using a Transcriptor first-strand cDNA synthesis kit (Roche Diagnostics GmbH, Mannheim, Germany) according to the protocol provided by the manufacturer. The cDNA obtained in this way was stored at −20°C until further analysis.

### RT-qPCR.

The RT-qPCR was performed using the previously designed primers (Table 4). The RT-qPCR was designed and performed on a LightCycler 480 instrument II (Roche Diagnostics GmbH) using the LightCycler 480 SYBR green I master reagent kit (Roche Diagnostics GmbH) according to the manufacturer's protocol.

**TABLE 4:** Primers used in the research

Strain and primer type	Primer sequence	References
RHDV		
Forward	5′-AAATAGTGGGACTKCAACCAGTACCT-3′	34 – 36
Reverse	3′-GGAGATRGGGTTGTCRAYTGCAGAC-5′	
RHDV-2		
Forward	5′-TGGAACTTGGCTTGAGTGTTGA-3′	36, 37
Reverse	3′-ACAAGCGTGCTTGTGGACGG-5′	

The trials were divided into two groups. The first group was subjected to qualitative PCR with the use of primers specific for classic RHDV strains (L. europaeus GI.1), while primers specific for RHDV2 (L. europaeus GI.2) were used for the second group. All reactions were performed in triplicate. In addition, a series of dilutions of full-length L. europaeus GI.2 transcript standards were prepared, ranging from 1 × 10^6^ copies/μL to 1 × 10^2^ copies/μL. A curve was then prepared using a LightCycler 480 instrument II (Roche Diagnostics GmbH) and used for absolute quantitative analysis to determine the copy numbers of L. europaeus GI.2.

### Sequencing and phylogenetic analysis.

A 320-bp fragment of the capsid protein VP60 gene region was sequenced with the primers OIE-F (5′-CCTGTTACCATCACCATGCC-3′) and OIE-R (5′-AACCCTCCAGGTACTGGTTG-3′) (32). Sequencing was performed in both orientations using the BigDye Terminator v1.1 cycle sequencing kit (Applied Biosystems, Foster City, CA, USA) and the SeqStudio genetic analyzer (Applied Biosystems). For phylogenetic analysis of the partial VP60 sequences, the maximum likelihood method was applied using MEGA7 software (33).

### Data availability.

The sequence data have been submitted to GenBank under accession numbers ON548890 to ON548897.
